# Is the increase in oil pollution a possibility of the presence of diverse microorganisms? An experimental dataset on oil prevalent areas of Goa, India

**DOI:** 10.1016/j.dib.2016.07.048

**Published:** 2016-08-17

**Authors:** Bhagwan N. Rekadwad, Chandrahaysa N. Khobragade

**Affiliations:** School of Life Sciences, Swami Ramanand Teerth Marathwada University, Nanded, India

**Keywords:** Bacterial pigments, Goan beaches, Hydrocarbon resistant bacteria, Oil and tar pollution, Microbial diversity

## Abstract

Survey data and wet lab reports presented in this paper were collected from Western coastlines of India from Goan beaches. Oil polluted areas were captured on camera as evidence for oil and tar pollution. Several microorganisms showing diverse characteristics such as pigment producers, salt tolerant and hydrocarbon resistance were isolated and cultured in the laboratory. The dataset presented in this paper supports “A case study on effects of oil spills and tar-ball pollution on beaches of Goa (India)” (Rekadwad and Khobragade, 2015) [1] and “Microbial diversity of oil spills and tar resistant bacteria isolated from beaches of Goa (India)” (Rekadwad and Khobragade, 2016) [2].

**Specifications Table**

**Table:** 

Subject area	*Life Sciences*
More specific subject area	*Environmental Microbiology*
Type of data	*Figures; Videos*
How data was acquired	*Through field work, survey and wet laboratory work*
Data format	*Raw*
Experimental factors	*Investigation of oil and tar polluted areas, isolation of hydrocarbon resistant microorganisms.*
Experimental features	*Oil pollution evidences were recorded from Colva beach to Arambol beach in Goa (South to North Goa). Oil contaminated samples used for isolation of microorganisms at the environmental temperature present at the time of sample collection.*
Data source location	*Goa coastline, India*
Data accessibility	*Data is available within this article.*

The following is Supplementary material related to this article Video 1, Video 2.

Video 1 Arambol beach oil pollution: offshore oil spill impact.

Video 2 Arambol beach oil pollution: shoreline oil spill impact..

**Value of the data**

• This data could be used to identify and study the extent of the impact of oil pollution.
• Data presented in this article could be used to study effects of oil pollution on foreshore and backshore of the polluted coastal regions.
• Microorganisms isolated in this study would have potential in bioremediation of tar-ball deposition on the seashore, Goan beaches, and other oil-polluted sites.

## 1 Data

**Fig. 1:**
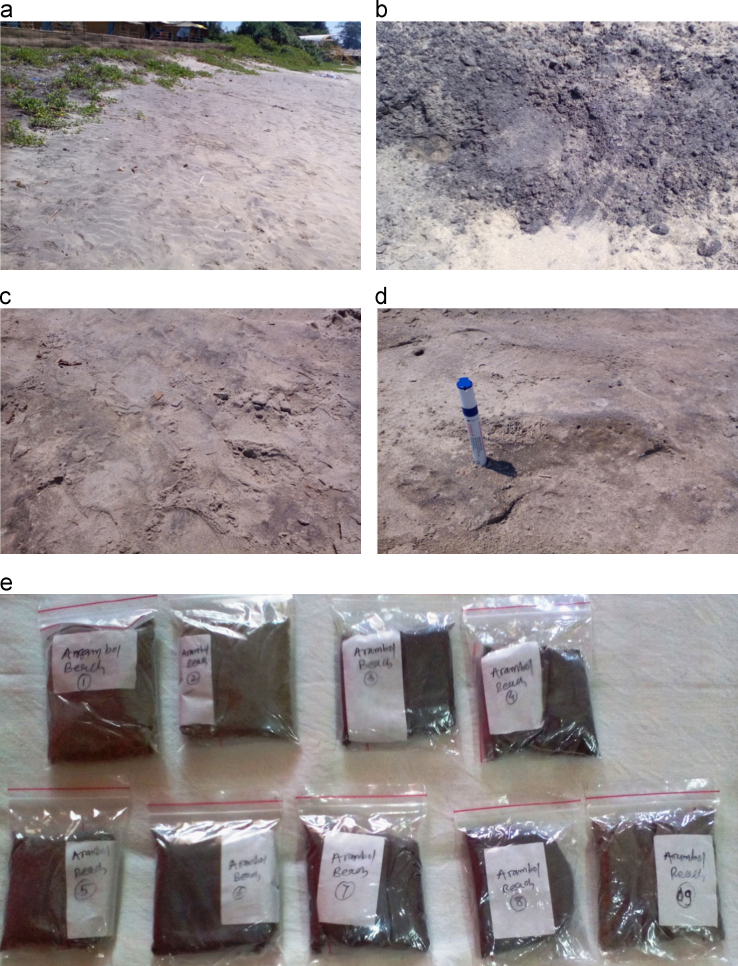
(a–e) Sand on oil and tar polluted Arambol beach was stained and appeared blackish in color.

**Fig. 2:**
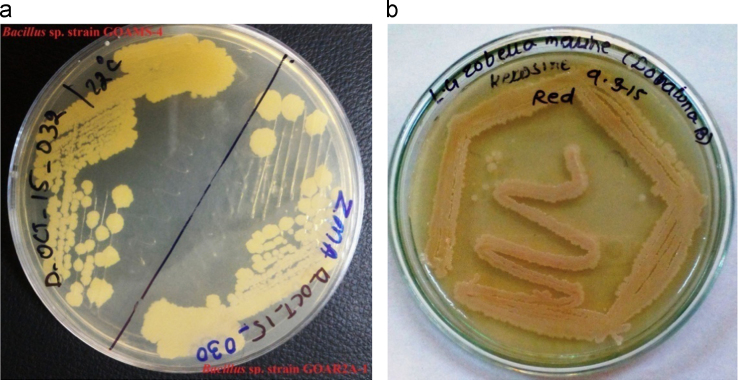
Salt tolerant, hydrocarbon resistant and pigment producing microorganisms isolated from Arambol beach sand (a) and Dona Paula beach sand (b).

Data include evidence of oil spills and tar-ball pollution on the coastal ecosystem of Goa. Data of diverse microorganisms isolated from the oil contaminated samples tabulated and figured in the understandable form [1], [2]. In Fig. 1 evidence of oil polluted beach capture in camera. In Fig. 2 diverse microorganisms isolated from oil polluted sand of Arambol and Dona Paula beaches were cultured in the laboratory.

## 2 Experimental design, materials and methods

Extensive study and field work were performed for collection of data on oil spills and tar pollution on Goan beaches from Margao (Colva beach) to Arambol (near Maharashtra border). The flight distance between above two places is approximately 52 km. Questionnaire and oral interviews were the important tools used for gathering information on oil spills and tar-ball pollution prevalent areas. Composite sampling, stratified sampling, grab sampling and accident sampling methods [3], [4], [5], [6], [7], [8] were used for collection of oil stained sand, soil and polluted water samples. Collected samples were refrigerated immediately in ice box after collection until use. Microorganisms isolated in the laboratory using Zobell Marine agar, R2A medium, Mannitol salt agar and Blood agar medium [9], [10], [11], [12].
